# Student-led peer review of an online teaching file: perspectives after 2 years

**DOI:** 10.1080/10872981.2020.1843356

**Published:** 2020-11-30

**Authors:** Bryan R. Bozung, Kaiulani Houston, John F. Lilly, Sheryl G. Jordan, Lynn A. Fordham, Gary Beck Dallaghan

**Affiliations:** a University of North Carolina School of Medicine, Chapel Hill, NC, USA; b UNC Health Care, Chapel Hill, NC, USA; c University of North Carolina School of Medicine Department of Radiology, Chapel Hill, NC, USA; d University of North Carolina School of Medicine Office of Medical Education, Chapel Hill, NC, USA

**Keywords:** Peer review, student editor, peer teaching, near peer learning, teaching file, case-based, medical student

## Abstract

**Problem:**

Opportunities for self-directed learning were missing from our medical school curriculum in general and on our radiology electives specifically. Our objective was to explore the feasibility and benefits of using medical students in the development of our student-created teaching files.

**Approach:**

In 2018, a website was developed at our institution to support medical student radiology education and create a repository for the online publication of student-developed teaching cases. Students participating in radiology clerkships at our institution had an opportunity to submit case presentations for publication to our online teaching file following peer review. The medical students participated in the peer review process facilitated by the faculty director of radiology undergraduate medical education. The faculty member oversaw the training of new student editors and the development of a peer review guide.

**Outcomes:**

The peer review guide included goals of the teaching file project and direction regarding the peer review process. Student editors were trained using the peer review guide in conjunction with individual meetings with the faculty mentor. At twenty-four months, 82 student-developed cases had been published to the online teaching file following medical student peer review. The teaching file had garnered 3884 page views.

**Next Steps:**

The medical student-led peer review process meets core competencies in self-directed learning. The authors plan to explore the application of peer-assisted learning theories to the editing and peer-review process.

## Introduction

Self-directed learning has been shown to be effective in health sciences education [1–3]. Formal opportunities for self-directed learning take many forms, such as peer instruction [4], case-based learning [5], flipped classrooms [6], and/or digital resources [7,8]. In preparing for a recent accreditation visit, a gap was noted in the medicals school curriculum. There were no formal opportunities for self-directed learning.

In 2018, a cloud-based website platform was developed to support medical student radiology education. This allowed us to create a repository for online educational materials. Of the online materials, students identified topics they were interested in learning and submitted cases for publication, which are referred to as ‘teaching files’. The intent of the teaching files is to provide medical students resources to direct their learning based on self-identified gaps in knowledge.

We previously reported the usage of the case files at 6 months and included data from a survey analyzing student impressions of the educational intervention[9]. The initial study examined the feasibility, establishment, and effectiveness of a publicly accessible student-produced case-based teaching file.

As part of the implementation of the teaching files, medical students acted as peer reviewers of new content. This experience increased opportunities to engage in self-directed learning and peer-assisted learning. This article focuses on the student-led peer review process employed in the creation of the teaching file.

## Approach

### Case development

The establishment of the website and survey of students’ impressions of the educational intervention were described in detail by Lilly et al [9]. In summary, the Department of Radiology created a medical student-centric radiology website with pertinent radiology educational resources, faculty-curated course-specific content, and the teaching file comprised of student-created de-identified cases organized by radiology subspecialty and listed by primary diagnosis. Page views for the website were recorded in real time.

Radiology clerkships at our institution typically require a formal presentation of a case identified by students while on rotation. Students had the option to refine and submit their case for online publication following peer review by medical student editors. In addition to the clinical background, imaging, findings, and interpretation often found in cases, students were asked to include an evaluation of cost and appropriateness of imaging decisions using American College of Radiology (ACR) Appropriateness Criteria® [10].

### Student peer review process

Case submission, peer review, and publication were overseen by the faculty director of radiology undergraduate medical education. The faculty member facilitated a double-blind peer review process by de-identifying each case submission before distribution to a student editor; receiving peer reviewer feedback, corrections, and opinion on whether or not to publish the case; and providing the peer feedback to the author. The faculty member also reviewed the case, providing feedback and corrections as necessary.

### Student editor

At the project outset, one student performed the role of peer editor. That student worked with the faculty advisor to develop the process for peer review and create a peer review guide to train future student editors. He continued the peer reviewer role in a transitional manner into his intern year, during which time he passed the responsibility to new student editors. New editors were selected from medical students who had engaged with radiology faculty in research projects and were in clinical training years.

The project was deemed exempt after review by the UNC Institutional Review Board (IRB Number 18–1308).

## Outcomes

The peer review guide, provided to new student editors, included goals of the teaching file project and individual teaching cases; instructions on providing feedback, maintaining a consistent layout, and emphasizing ACR Appropriateness Criteria®; and direction regarding academic integrity and ensuring citation of sources. New student editors also met individually with the faculty member to review the peer review guide and discuss questions about the peer review process. When there were multiple editors at one time, cases were evenly distributed and assigned with consideration of editors’ intended specialties.

Editors ensured presentations generally followed a recommended format, providing consistency throughout the teaching file. Peer reviewed cases approved for publication were then assigned to a subspecialty category and uploaded to the online teaching file. An ‘Emerging Knowledge’ category included student presentations that departed from standard case files and addressed broader ideas such as ethics and artificial intelligence in radiology.

After two years, four students acted as editors for the case-based teaching file – one during the first year of the project, and three co-editors during the second year. Of the four student editors, two chose radiology as a specialty, one neurology, and one general surgery. These peer editors each reviewed an average of 1–2 cases per month, though the number of case submissions varied throughout the year and correlated with student enrollment in radiology clerkships.

**Figure 1.**
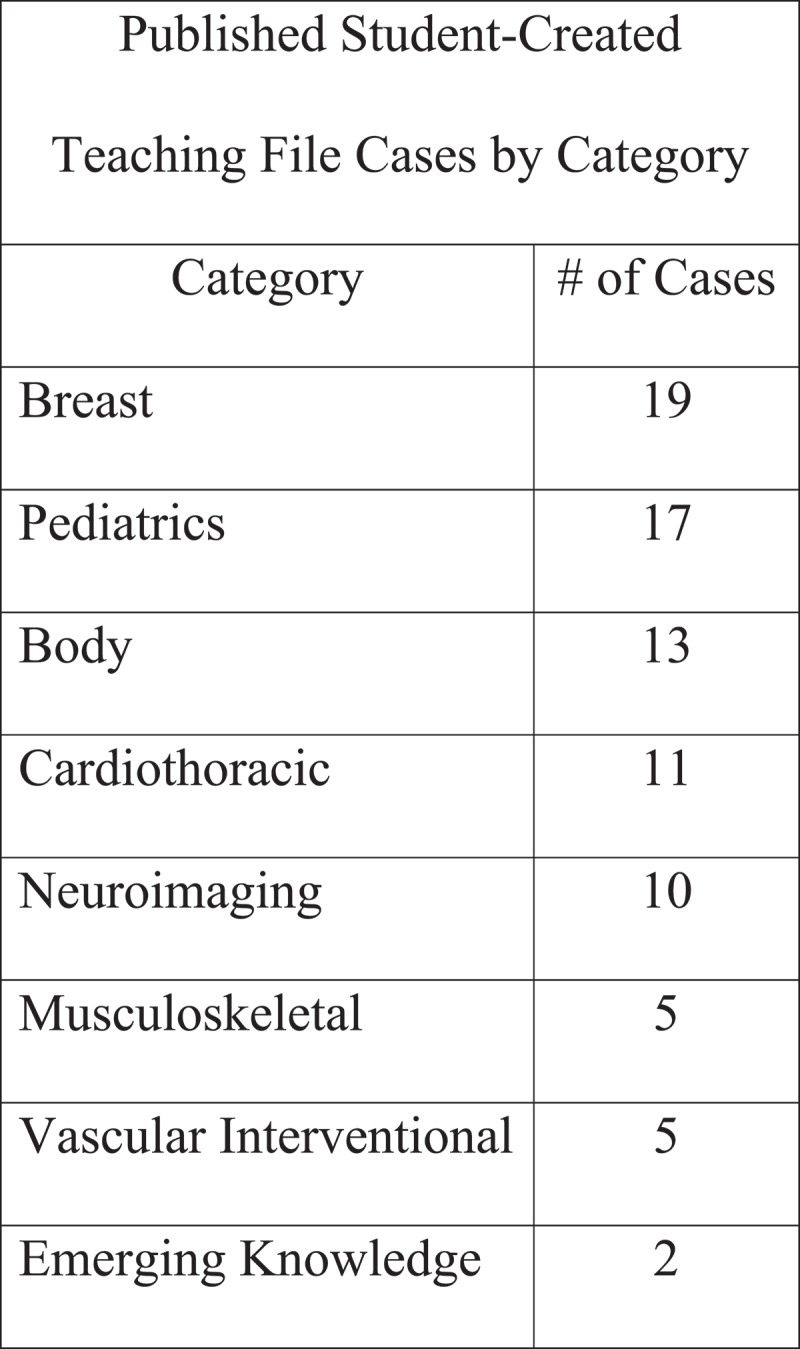
Student-developed cases published to the online teaching file following peer review at two years

Students seemed to be more interested in submitting cases during the spring and summer, which corresponded with the beginning of our institution’s ‘Individualization Phase’ when students could begin taking radiology clerkships but before interview season began in the fall. At two years, 82 student-developed cases had been published to the online teaching file following peer review (Figure 1).

The majority of student case submissions were already well-refined after being orally presented and often prescreened by the student’s clerkship director prior to submission for peer review and publication. The faculty member overseeing the double-blind peer review process also prescreened submissions for obvious deficiencies before sending cases to peer reviewers. She estimated 20% of submissions were returned to authors to be brought more into alignment with the instructions students had received for creating case presentations. Approximately one quarter of returned cases were never resubmitted. Cases that generally followed provided guidelines moved forward to peer review. Student editors proofread presentations and provided feedback to authors with requested – usually minor – changes. Student authors were consistently willing to accept and implement requested changes. Nearly all peer reviewed cases were ultimately accepted for publication, likely a result of the prescreen process.

Website analytics for the medical student radiology education site demonstrated 3884 page views for the teaching files.

### Perspectives from students who have acted as teaching file student editors

Editor #1: ‘As an acting student-editor of the student-authored teaching files for the last 12 months, I have witnessed a shift in the trajectory of my own medical education and that of my peers as a direct result of having access to the teaching files. In alignment with the surveyed data, I have utilized the teaching files in 3rd and 4th year clerkships for both self-directed learning and practice in gaining proficiency and comfort with incorporating radiologic evidence in developing differentials and treatment plans. I have referred students who have not taken a radiology clerkship to the site when asked about resources to increase their knowledge of ACR criteria and build their confidence with personally reviewing radiographic data. As a peer reviewer, I have edited interesting cases year-round in my desired specialty of neurology. This experience has expanded my own knowledge-base and given me the opportunity to teach others. During the COVID-19 pandemic, I referred to the teaching files as a way of adapting to the changes in access to traditional medical education. It is my expectation that these case-based teaching files will reach a wider student audience as medical school curricula incorporate virtual tools well-suited for the medical training of Millennials and Generation Z.’

Editor #2: ‘My anecdotal experience corroborates the data from the students surveyed before I participated in a radiology clerkship. The teaching file is a resource I frequently reference to review indications, risks and benefits, cost, and radiologic findings for specific diagnoses. Preparing my own case for submission was an experience in self-directed learning during which I discovered resources I have continued to use on subsequent rotations to better understand imaging appropriateness. While acting as an editor of the teaching file I have been continually impressed by the high quality of case submissions and the succinct yet effective teaching performed by my peers. I am pursuing radiology as a specialty and the student-developed teaching file has been a boon to my education that I expect to continue referencing during my training. The opportunity to act as a peer reviewer has been an important experience in self-directed learning and peer-assisted learning.’

## Discussion

One of the core principles applied in the creation of the teaching file was peer-assisted learning. Peer, or near-peer, learning has been demonstrated to have similar outcomes to faculty teaching, but the process of peer teaching helps the student teaching to develop knowledge, teaching ability, and professional skills [4,11–14]. Though peer teachers often underestimate the value of their teaching, learners generally appreciate peer teaching and rate the experience highly. Teaching file student editors engaged in bidirectional peer learning. As learners they benefited from case-based learning through review of peers’ carefully crafted case presentations [5]. As teachers they used their experience from radiology clerkships and as editors of the teaching file to provide constructive feedback and education in regards to professional case presentation and imaging appropriateness.

Medical student peer review promotes the development of core competencies in self-directed and lifelong learning [15]. Self-directed study has been shown to significantly increase knowledge acquisition in medical students [1,2]. Student editors’ roles require familiarizing themselves with topic matter when reviewing cases, including appropriateness criteria. Engaging in the process of peer review involves fundamental aspects of self-directed study and lifelong learning including ‘self-assessment of learning needs; independent identification, analysis, and synthesis of relevant information; appraisal of the credibility of information sources; and feedback on these skills’ [6,15]. The faculty member overseeing the peer review process provides feedback to student editors as needed. Both peer reviewers and students submitting cases benefit from participating in the process of publication.

The process of peer review and students’ creation of case-based teaching files promote competency in systems-based practice, with emphasis placed on ACR Appropriateness Criteria®, cost considerations, and communication regarding imaging rather than focusing primarily on interpretation of radiological studies [16]. The 6-month survey results demonstrated increased student confidence in these areas after reviewing teaching file cases, fostering increased preparedness to navigate appropriate use of imaging in a larger system [9]. Though not directly surveyed, similar benefits are expected for student editors with additional experience navigating the role of a peer reviewer in a system of publication. The teaching file also involves blended learning as a self-directed study tool and as a resource that attendings can use for teaching or referring students to [3,6]. It utilizes technology as an online, open access tool readily accessible for review on demand – important for millennial learners used to having information accessible within a few taps [7,8].

### Limitations

Radiology is a specialty heavily reliant on technology and is well-positioned to be a pioneer in digital medical education from case files to virtual reality simulations. However, any specialty or institution could create a student-driven teaching file similar to ours. One of the great benefits of the teaching file has been the opportunity for students to act as peer reviewers.

We are exploring additional ways to use our teaching file. Sheng et al. demonstrated another effective method of teaching imaging appropriateness by assigning small groups of students to create cases specific to their intended specialties [17]. Their use of small groups and specialty-specific cases are excellent examples of additional methods that could be applied in the development of student-made teaching files.

Peer review could also be expanded. Separate from peer review during the publication process, students on clerkships could benefit from a requirement to review peers’ presentations prior to formal oral presentations at the end of the clerkship. From the authors’ experience, these types of peer review exercises are more common in undergraduate and even secondary school settings, but less utilized in medical school when such an experience may be even more pertinent for professional development.

## Conclusions

The medical student-led peer review process, like the open access website and student-developed online teaching file, successfully meets core competencies in self-directed and lifelong learning and takes advantage of research-driven educational methods.
